# Fish Meal Replacement in Chum Salmon (*Oncorhynchus keta*) Diet With Alternative Protein Sources

**DOI:** 10.1155/anu/4630480

**Published:** 2025-05-06

**Authors:** Buddhi E. Gunathilaka, Geun-Up Kim, Sang-Min Lee

**Affiliations:** ^1^Department of Aquatic Life Medicine, Gangneung-Wonju National University, Gangneung, Republic of Korea; ^2^Gangwon Sea Grant, Gangneung-Wonju National University, Gangneung, Republic of Korea

**Keywords:** alternative protein sources, chicken byproduct meal, chum salmon, fish meal replacement, meat meal, muscle quality, Pacific salmon

## Abstract

Chum salmon (*Oncorhynchus keta*) is an indigenous salmonid species found in Korea. This experiment was conducted to evaluate the effects of fish meal (FM) replacement with krill meal (KM), soy protein concentrate (SPC), meat meal (MM), and chicken byproduct meal (CBM) in chum salmon diets. A control diet was designed to contain 60% FM, 5% KM, and 8% SPC (FM60). Three diets were formulated to contain 45%, 30%, and 15% FM (FM45, FM30, and FM15). The reduced protein levels after FM replacement were supplied with a mixture of KM, SPC, MM, and CBM. Fish, averaging 5.94 ± 0.19 g, were fed four experimental diets or a commercial diet (COMF) for 6 weeks. Final body weight of fish fed FM30 and FM15 diets were significantly increased than fish fed COMF. Feed intake (FI) was significantly higher in FM60, FM45, and FM30 groups than COMF group. Condition factor (CF) was significantly higher in FM15 group compared to FM60 and COMF groups. Muscle saturated, highly unsaturated, and omega-3 fatty acids were significantly higher in COMF group compared to those of fish fed other diets. FM15 groups exhibited significantly lower EPA and DHA levels compared to FM60, FM45, and FM30 groups and significantly higher omega-6 levels compared to other groups. The results indicate that a mixture of KM, SPC, MM, and CBM can be used to replace FM in chum salmon diet down to 30%–15% while maintaining normal performance compared to diet containing 60% FM.

## 1. Introduction

Chum salmon (*Oncorhynchus keta*), also known as dog, keta, or silverbrite salmon, is a species of Pacific salmon (*Oncorhynchus spp*.) native to the coastal rivers of the North Pacific [1]. Chum salmon naturally migrate to Korea, Japan, North America, and Russia, habituating in a wide range. Chum salmon is the second largest of capturing salmon species in the North Pacific Ocean [2]. It is the only indigenous salmonid species found in Korea [3, 4]. Chum salmon was hatched and released to natural habitats by artificial hatcheries to maintain its population. However, the return of fish is decreasing compared to the number of released juveniles due to several factors, including global warming in the last two decades [5, 6]. Pacific salmon possess zooplankton and nekton feeding behaviors in the sea. Chum salmon is one of the species feeding on zooplankton [7]. It also feeds on copepods and euphausiids in addition to gelatinous zooplankton when competitive species are abundant in the water [8]. It migrates to saltwater in early development stages and is widely distributed due to the high diversity of prey.

Korean chum salmon exhibits a lower genetic diversity and unique genetic integrity [9]. Therefore, it is an important species that should be conserved with high priority. It is also a potential aquaculture species to increase domestic salmon production and to reduce imports, as the quantity and cost of imports were increasing yearly [10]. In this regard, a number of studies were conducted during the last few decades to understand the distribution, morphological characteristics, genetic diversity, behavior patterns, physiology, and diseases of chum salmon migrate to Korea [3, 11–13]. As a part of these studies, Cho et al. [14] reported that proper feeding frequency was two times a day for chum salmon in a recirculating aquaculture system.

Salmon diets were usually formulated with high levels of fish meal (FM) as main protein source [15]. Alternative protein sources have been used to replace FM in salmon diets during the last two decades due to increased fish production and limitation of FM supply [16, 17]. Consequently, different types of protein sources were used to reduce FM levels in diets for Pacific salmon species. In the present study, we evaluated krill meal (KM), meat meal (MM), chicken byproduct meal (CBM), and soy protein concentrate (SPC) as alternative protein sources to replace FM. These ingredients were well studied, and their usability to substitute FM was reported in different fish species [18, 19]. MM and CBM contain high protein levels as suitable alternative animal-based protein sources for fish diets [18, 20]. SPC was also reported to contain favorable protein for fish, as well studied ingredient in FM-replaced diets [21]. KM provides marine-originated protein and lipids as an ingredient produced using marine crustacean species [22]. Several studies were conducted in the literature to evaluate some alternative protein sources in Pacific salmon diets, including these protein sources. For instance, dietary KM improved the growth performance of chum salmon when 5% KM was supplemented to the diet containing 65% FM [23]. Dietary supplementation of KM and Pacific hake (*Merluccius productus*) hydrolysates mitigated adverse effects of soybean meal on Chinook salmon [24]. Doughty et al. [25] observed reduced growth performance in Chinook salmon (*O. tshawytscha*) fed diets containing 15% FM, poultry byproduct meal and corn gluten meal. Growth performance of coho salmon was not adversely affected when 20% of FM was replaced with poultry byproduct meal [26]. FM level in coho salmon diet was reduced from 60% to 50% with SPC without sacrificing growth performance and feed efficiency (FE) [21]. Therefore, these previous studies indicate the possibility of FM replacement in pacific salmon diets using these protein sources. However, species-specific studies are required to investigate nutrient requirements of Pacific salmon in line with their natural feeding habits [27]. Nutritional studies on other pink salmon (*O. gorbuscha*), sockeye salmon (*O. nerka*), and chum salmon were rarely conducted. In the case of chum salmon, limited studies were conducted a few decades ago to investigate silkworm pupae powder, dried beef liver, KM, and earthworm powder as single FM replacers [23] and zooplankton [28] in their diets.

The present study was designed to understand and reveal the nutritional data of chum salmon. We investigated an efficient FM level in diet containing KM, MM, CBM, and SPC through the effects on growth performance, feed utilization, innate immunity, biochemical parameters, and muscle composition of chum salmon, while investigating supplementary effects of the alternative protein sources mixture in diets containing graded levels of FM.

## 2. Materials and Methods

### 2.1. Experimental Diets

**Figure 1:**
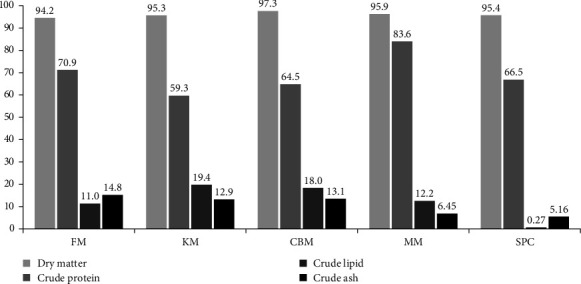
Proximate composition (dry matter basis [%]) of fish meal (FM), krill meal (KM), chicken byproduct meal (CBM), meat meal (MM), and soy protein concentrate (SPC).

Four experimental diets were formulated to contain approximately 52% crude protein and 21% crude lipid, as shown in Table 1. A control diet was designed to contain 60% FM, 5% KM, and 8% SPC (FM60). Three other experimental diets were formulated to contain 45%, 30%, and 15% FM (named as FM45, FM30, and FM15) and increasing levels of KM, SPC, MM, and CBM at 1:1:1:1 ratio. The inclusion levels of KM, SPC, MM, and CBM were increased as 26%, 40%, and 54% to compensate for reduced FM protein in FM45, FM30, and FM15 diets, respectively. Proximate composition of protein sources was illustrated in Figure 1. All the diets were added with 2% squid liver powder, 1.2% corn gluten, and 8% wheat flour as protein-containing ingredients. FM replaced diets were added with lysine, methionine, and monocalcium phosphate to compensate for reduced amount after FM replacements. The weight of all dry ingredients was measured according to feed formulation, added with 13% fish oil and 30% distilled water before mixing to make the dough. Then, the dough was passed through a noodle machine (SP-50, Gum Gang Engineering, Korea), crushed into an ideal size (2–5 mm), and dried at 40°C for 12 h dry diets were stored at −20°C. Amino acid and fatty acid profiles of experimental diets are presented in Tables 2 and 3, respectively.

### 2.2. Feeding Trial and Experimental Conditions

Chum salmon was provided by FIRA, Yangyang, South Korea, and feeding trial was conducted in the fish rearing facility at the Marine Biology Center of Gangneung-Wonju National University. They were fed a commercial diet (COMF) and acclimatized in three fiber-glass circular tanks for 2 weeks. Chum salmon, 750 individuals averaging 5.94 ± 0.19 g, were placed in each of 15 fiberglass tanks having a 300 L capacity in a recirculatory system at a density of 50 fish. Tanks were randomly assigned to three replicates of four formulated diets and the COMF. Then, fish in each tank were daily fed assigned diets until satiation at 09:00, 13.30, and 17:00 h for 6 weeks. Feed intake (FI) was recorded daily. The photoperiod was controlled with fluorescent lights to provide the natural day length. Water temperature was maintained around 12°C using a seawater cooler (DA-1500L, Daeil Co.Ltd., Busan, Korea) attached to the recirculatory system. Also, the salinity, pH, and dissolved oxygen level were monitored every day and maintained as 32.2 ± 0.4 ppt, 7.84 ± 0.27, and 7.12 ± 0.31 mg/L, respectively, during the feeding trial. There were no notable differences in water quality parameters among experimental tanks.

### 2.3. Sample Collection and Analysis

Fish in each experimental tank were starved for 24 h at the end of the 6-week feeding trial. Then, fish were caught carefully with fish-catching net while counting. The bulk fish weight of each tank was measured for the calculation of weight gain (WG), specific growth rate (SGR), FI, FE, and protein efficiency ratio (PER). Twelve fish from each tank were randomly selected and anesthetized with 2-phenoxyethanol (200 ppm). Blood samples were collected from the caudal vein of 12 captured fish. Blood samples from six fish were withdrawn to separate plasma for biochemical analyses, and from the remaining six fish to separate serum samples for immune parameter analyses. Blood samples were withdrawn with heparinized syringes to prevent clotting before separated into plasma. Plasma and serum samples were separated by centrifugation at 5000 *g* for 10 min, pipetted out to new vials and stored at −80°C. Blood was kept at room temperature for 30 min to facilitate clotting before separating serum samples. The remaining 12 fish after blood sampling were stored to freeze at −20°C for proximate composition analyses of muscle, amino acids, and fatty acid levels. Then, the remaining fish in each tank were killed with a high dose of 2-phenoxyethanol (500 ppm). The weight and length of each fish were measured to determine condition factor (CF). Each fish was dissected, and viscera and liver were collected to measure the weight for calculation of hepatosomatic (HSI) and viscerosomatic indices (VSI).

Moisture and ash levels of the feed ingredients, diets, and chum salmon muscle were analyzed after drying at 125°C for 6 h in a dry oven and at 550°C for 6 h in a muffle furnace, respectively, according to standard methods as explained in AOAC [29]. Crude protein level was analyzed using 0.5 g of samples after digesting with concentrated sulfuric acid (95%), distilling with 8M sodium hydroxide, recovering nitrogen with boric acid, and titrating with 0.05M sulfuric acid with a Kjeltec Analyzer (Buchi, Switzerland). Crude lipid level of samples was determined after boiling 0.5 g of each sample in 60 mL ethyl ether in a Soxhlet extractor (VELP Scientifica, Italy). Muscle and diet fatty acid profiles were determined using a gas chromatographic method, as followed by Sankian et al. [30] and calculated as a percentage of the total fatty acid level in each sample. Amino acid composition of both diet and muscle samples was analyzed after properly controlled acid hydrolysis (6 N HCL reflux for 23 h at 110 °C) using high-speed Amino Acid Analyzer (L-8800, Hitachi, Tokyo, Japan) at the Marine Bio Regional Center, Gangnang, Korea. Lysozyme activity was measured after mixing 20 μL of serum samples with 100 μL of lyophilized *Micrococcus lysodeikticus* (Sigma, St. Louis, USA) as a substrate based on a turbidimetric technique following Khosravi et al. [31]. Serum superoxide dismutase (SOD) activity was measured in 20 μL of serum samples by analyzing inhibition rate of tetrazolium with SOD enzyme in serum using a commercial assay kit (19,160, Sigma, USA). Plasma biochemical parameters, glutamic–oxaloacetic transaminase (GOT), glutamate pyruvate transaminase (GPT), alkaline phosphate (ALP), total protein (TP), triglyceride (TG), glucose (GLU), and total cholesterol (TCHO) levels, were measured with an automated blood analyzer (FUJI DRI-CHEM NX500i, FUJIFILM, Japan) equipped with DRI-CHEM slides purchased from FUJIFILM Co., Japan.

### 2.4. Statistical Analysis

The data were analyzed using one-way analysis of variance. The significance of differences in the mean effects of diets were determined using Duncan's [32] multiple range test with SPSS version 20.0 (SPSS Inc., Chicago, IL). Shapiro–Wilk's and Levene's tests were applied for the verification of homogeneity of observed data. The statistical significance of data was determined at *p* < 0.05. Data were presented as mean ± standard error (SE). Statistical analysis of percentage data was performed after arcsine transformation.

## 3. Results

### 3.1. Growth Performance and Feed Utilization

Results of growth performance and feed utilization are shown in Table 4. The final body weight of the fish fed FM30 and FM15 diets were significantly higher than that of fish fed COMF (*p* < 0.05). FI of FM60, FM45, and FM30 groups were significantly higher than that of fish fed COMF (*p* < 0.05). WG and SGR were not significantly affected by experimental diets. However, fish fed FM30 and FM15 diets exhibited high WG and SGR compared to other diets, although the observed values were not significantly higher according to statistical analysis. The lowest WG and SGR were observed in fish fed COMF. FE, PER, and survival were also not significantly affected by dietary treatments.

### 3.2. Nonspecific Immune Response

Lysozyme and SOD activities of fish fed experimental diets are shown in Table 5. Both parameters were significantly unaffected by the dietary supplementation of alternative protein sources in the pace of FM.

### 3.3. Plasma Biochemical Parameters

Biochemical parameters of chum salmon fed experimental diets are presented in Table 6. GPT, GOT, ALP, T-CHO, TG, TP, and GLU levels were significantly unaffected by the experimental diets.

### 3.4. Biometric Parameters

Biometric parameters of chum salmon fed experimental diets are presented in Table 7. CF was significantly higher in FM15 group compared to that of FM60 and COMF groups (*p* < 0.05). HSI and VSI were not significantly affected by the experimental diet.

### 3.5. Muscle Proximate Composition

Muscle proximate composition of chum salmon fed experimental diets is shown in Table 8. Muscle dry matter, protein, lipid, or ash compositions were not significantly affected by the FM replacement or supplementation of alternative protein sources in diets.

### 3.6. Muscle Fatty Acid Profiles

Muscle fatty acid profile of fish fed experimental diets is shown in Table 9. Total saturated, omega-3, and HUFA levels were significantly higher in COMF group compared to those of fish fed other diets (*p* < 0.05). EPA (20:5 n-3) and DHA (22:6 n-3) levels were also significantly higher in COMF group compared to other diets (*p* < 0.05). FM15 groups exhibited significantly lower EPA and DHA levels compared to FM60, FM45, and FM30 groups (*p* > 0.05). However, omega-6 fatty acid level was significantly lower in COMF group compared to all other groups (*p* > 0.05). FM15 group exhibited significantly higher omega-6 level compared to FM60, FM45, and FM30 groups (*p* < 0.05).

### 3.7. Muscle Amino Acid Composition

Muscle amino acid profile of fish fed experimental diets is shown in Table 10 as a percentage of total amino acid level in each diet. Muscle essential and nonessential amino acid levels were significantly unaffected by dietary treatments.

## 4. Discussion

Growth performance of chum salmon was not significantly affected by the formulated feed in the present study, indicating that FM replacement and supplementation of alternative ingredients had no adverse effects on fish growth. However, FBW of fish fed COMF diet was significantly lower than that of FM30 and FM15 groups. A number of factors influence the growth of fish. Protein and lipid ratios influenced the growth performance of salmon [33, 34]. In the present study, there were considerable differences in dietary lipid levels and fatty acid composition of COMF, and formulated feeds. Therefore, lower crude lipid levels in COMF might result in low growth performance in the present study. The unknown feed ingredients in the COMF might also be a reason for the differences observed in chum salmon, as the ingredient types also affect the growth performance of salmon [35, 36]. However, results of the present study obviously indicated that FM replacement in diets down to 15% with KM, SPC, CBM, and MM had no adverse effect on growth performance of chum salmon because WG and SGR were slightly higher in FM30 and FM15 groups compared to those of other groups. According to literature, approximately 17% FM in coho salmon diet was replaced by poultry byproduct meal [26]. KM reduced FM level to 25% in Atlantic salmon (*Salmo salar*) diet without adverse effects on the growth performance [37]. FM level in coho salmon diet was reduced by 20% with SPC [21]. According to these studies [26, 37], high dietary CBM and KM retarded the growth performance of salmon. In the present study, experimental diets contained 13.5% of each alternative protein source even after reducing FM level to 15%. Therefore, negative impact expected from high level of single protein sources might not happen because a combination of these ingredients were used for FM replacement in chum salmon diet. Amino acid profiles of diets were not significantly changed after FM replacement because lysine and methionine, known as limiting amino acids, were included in low FM diets in the present study. Fatty acid profiles of diets were also not significantly affected although oleic acid (18:1 n-9) level was increased and omega-3 level was decreased with FM replacement. KM contains considerable levels of EPA and DHA [38]. Therefore, depleting levels of EPA and DHA after FM replacement might be compensated by KM to reduce impact of FM replacement. We also formulated diets to contain taurine as growth enhancer [39] and monocalcium phosphate to provide calcium and phosphorus [40], which were declined after FM replacement. Growth performance of chum salmon might be maintained in FM-replaced diet to match with high FM diet in the present study due to these reasons. Different dietary lipid levels should be investigated in future studies to evaluate their effects on growth performance of chum salmon.

Feed utilization was not suppressed by FM replacement or supplementation of KM, SPC, CBM, and MM. FI of COMF group was significantly lower than that of FM60, FM45, and FM30 groups. The differences observed in FI were in line with the growth performance, as the COMF group exhibited low final weight, resulting in significantly unaffected FE. According to literature, feed utilization of salmon was reduced after FM replacement in diets. For instance, partial FM replacement in coho salmon diet resulted in reduced feed utilization [26]. Dietary FM replacement with KM in Atlantic salmon diet also resulted in decreasing FE, although the values were not significantly different [37]. Both reports indicate that FM level can be replaced to some extent with a single alternative protein source. The results of the present study indicated that mixture of protein sources can be efficient for chum salmon compared to previous studies on other salmon species. Salmon species feed on a wide range of prey in their natural habitat [7]. Digestive system of chum salmon is comparatively larger than other Pacific salmon species [41, 42], indicating the efficient digestion of food from different sources. Therefore, different types of protein sources might be efficiently utilized by chum salmon in the present study, as observed in other fish species fed mixture of protein sources [18].

Lysozyme and SOD activities of chum salmon were not significantly affected by the dietary treatments, indicating that dietary FM can be replaced with KM, SPC, CMB, and MM without negative impact on immunity. Liver SOD activity of coho salmon was significantly decreased, when FM level in diets was reduced with poultry byproduct meal [26] or soybean meal [43]. However, Zhang et al. [42] observed that fermented soybean meal can reduce FM level to 25% without sacrificing liver SOD activity in coho salmon. Lysozyme activity of Atlantic salmon was not decreased when their diets contained 30% FM with poultry feather meal and lupin meal [43]. In contrast, Jo et al. [44] observed significantly improved both SOD and lysozyme activities in rainbow trout (*Oncorhynchus mykiss*) fed FM replaced diets containing a mixture of poultry byproduct meal, wheat gluten, blood meal, and shrimp soluble extract. They also observed comparable lysozyme activity to control group containing 42% FM when high level of FM was replaced. According to these studies, both lysozyme and SOD activities were not reduced when diets contained more than one alternative protein source. However, FM level in diets was not lower than 20% in all these studies. Therefore, it seems like the type and amount of protein sources in diet were important in maintaining lysozyme and SOD activities of salmon. In the present study, both activities were not reduced even after feeding a diet containing 15% FM (FM15). Natural food habit of chum salmon consists of a wide range of prey, including mollusks, squids, and crustaceans in seawater. Therefore, the ability to utilize a mixture of protein sources might be higher resulting in unaffected growth and immune status after feeding low FM diets. Fatty acid composition of experimental diets was changed when alternative protein sources were included. Omega-3 level was decreased when the FM level was decreased. Present results indicate that levels of dietary fatty acids in the present study had no influence on immune status of chum salmon in addition to the growth performance. However, the trial period is a limiting factor in the present study, although the WG percentage was approximately 150%. The fish size was also small, and fish were just after transforming to sea water. We assumed that further studies should be conducted for a long trial period using fish in different growth stages to investigate these assumptions.

Plasma biochemical parameters were also not significantly affected by FM replacement or addition of alternative protein sources in diets. Plasma biochemical indices represent the health and physiological status of organisms [45]. Hematological and biochemical parameters of Chinook salmon were reported to vary in freshwater and seawater stages. Fish size was also a reason for differences in these parameters [46]. Xu et al. [47] reported that biochemical parameters of coho salmon were affected with vitamin C level in diets due to its involvement in metabolic processes (i.e., lipid metabolism). Their results indicate that direct influences on metabolism can change the biochemical indices. Serum biochemical parameters of coho salmon were significantly influenced after partial replacement of FM in diets with poultry byproduct meal [26]. They assumed that lower nutrient content in plasma and increased damage occurred in hepatocytes were reasons for reduced biochemical indices in fish fed high poultry byproduct meal. However, biochemical parameters of Atlantic salmon were not significantly changed even after total FM replacement with KM, indicating that metabolic processes were not significantly changed due to KM [37]. Metochis et al. [48] observed no significant effects on serum TP and GLU levels of Atlantic salmon fed high SPC diets. Therefore, metabolic activities of salmon might not be changed significantly by SPC. In the present study, metabolic process of chum salmon might not be changed by the mixture of KM, CBM, MM, and SPC even after reducing FM level to 15%. Experimental diets in the present study contained comparable levels of amino acids, although fatty acids levels were different. However, omega-3 long-chain fatty acids did not change biochemical parameters of Atlantic salmon reared in cages [49]. Results of the present study were also in line with their observation. We assumed that different types and levels of nutrient provided by protein sources might alleviate the adverse effects of FM replacement on metabolic activities unaffecting biochemical indices of chum salmon during 6-week feeding trial.

Biometric parameters give a quantitative idea about the quality of fish including muscle gain, liver condition, and digestive efficiency [50]. CF represents the mass growth against body length. HSI and VSI represent liver and viscera weight as a ratio to body weight. CF of fish fed FM15 diet was significantly higher than that of FM60 and COMF groups, indicating high muscle gain in fish fed FM15 diet compared to length. The final weight of fish fed both FM30 and FM15 diets were significantly higher than the COMF group. However, CF of FM60 was also significantly lower than FM15 group, indicating that the fish length might be slightly higher in fish fed FM60 diet. Length is an important factor for determining fish growth, while CF represents the well-being of fish [51]. Therefore, long-term feeding trials should be conducted to evaluate the effects of dietary FM replacement with alternative protein sources on growth of chum salmon. In the present study, VSI and HSI were not significantly altered by the experimental diets. Muscle proximate composition of chum salmon was also not significantly affected due to FM replacement with protein sources, revealing that muscle fat deposition was similar in all groups. Formulated diets contained different fatty acid levels but comparable crude lipid levels. Muscle fatty acid composition of fish in present study reflected that of diet composition. A similar trend was observed in amino acid levels. Therefore, adipose fat deposition might not be significant in chum salmon fed FM replaced diets, indicating that nutrient digestion in diets might not be significantly influenced during the feeding trial.

Muscle fatty acid level was significantly affected by dietary treatments in the present study. COMF diet contained high SFA level, which was reflected in muscle samples. Omega-3 level was significantly lower in FM15 group compared to FM60, FM30 and FM45 groups. FM60, FM45, and FM30 groups contained comparable omega-3 levels. However, omega-3 level was gradually decreased when replacing FM from FM60 to FM30 diets without exhibiting significant differences. FM15 group exhibited a significantly lower omega-3 level compared to FM30 group, indicating that muscle composition of FM15 group was significantly manipulated due to nutrient composition in diet. An opposite trend was observed in omega-6 level as FM15 group exhibited significantly higher levels compared to FM60, FM45, and FM30 groups without reflecting dietary composition. Muscle HUFA level also shows a similar pattern although total HUFA amount was higher in muscle than diets compared to other fatty acids. In the present study, all formulated feeds contained approximately 20% crude lipid, while the COMF contained 7.5% crude lipid although muscle lipid and fatty acid levels were not significantly altered after the feeding trial. Huyben et al. [52] reported that carcass omega-3 level was increased in Atlantic salmon after feeding a high omega-3 diet compared to low omega-3 diets. Accordingly, we observed similar trends in muscle fatty acid levels in each group. They also reported that genes related to fatty acid synthesis were upregulated when diets contain low omega-3 levels with high lipid level or high omega-3 level with low lipid level, indicating that omega-3 requirement is related to the total lipid level in diets. The COMF diet used in the present study contained a higher omega-3 level and low lipid level compared to other diets. Accordingly, fatty acid metabolism might be accelerated in fish fed COMF diet. Further studies are required to estimate an optimum lipid level in chum salmon diets to improve muscle fatty acid profile. However, growth performance and immune status of fish was significantly reduced after decreasing dietary omega-3 level in salmon [53, 54]. In the present study, growth performance was reduced in fish fed COMF diet compared to other groups, indicating that fatty acid levels and total lipid level might play an important role to improve fish growth performance. Therefore, studies should be conducted to investigate the relationship between lipid level, lipid source, alternative protein source, and FM level in chum salmon diets to improve muscle fatty acid profile.

## 5. Conclusions

The results indicate that a mixture of KM, SPC, MM, and CBM can be used to replace FM in chum salmon diet down to 15%, while maintaining normal performance compared to diet containing 60% FM. Dietary FM level in between 30% and 15% with the mixture of KM, SPC, MM, and CBM seems to be sufficient for chum salmon to maintain a comparable muscle fatty acid profile to fish fed a diet containing 60% FM. However, low FM diets with different lipid levels and sources should be further investigated in chum salmon to improve the muscle fatty acid profile and evaluate its effects on overall performances. Long-term effects of low FM diets should also be investigated in future studies.

## Figures and Tables

**Table 1:** Formulation and proximate composition of the experimental diets for chum salmon (*Oncorhynchus keta*) (dry matter basis [%]).

Ingredients	FM60	FM45	FM30	FM15	COMF
Fish meal (sardine)^a^	60.00	45.00	30.00	15.00	—
Krill meal^b^	5.00	6.50	10.00	13.50	—
Soy protein concentrate^c^	8.00	6.50	10.00	13.50	—
Chicken byproduct^d^	—	6.50	10.00	13.50	—
Meat meal^e^	—	6.50	10.00	13.50	—
Squid liver powder	2.00	2.00	2.00	2.00	—
Corn gluten meal	1.20	1.20	1.20	1.20	—
Wheat flour	8.00	8.00	8.00	8.00	—
Fish oil	13.00	13.00	13.00	13.00	—
Mineral Mix^f^	1.00	1.00	1.00	1.00	—
Vitamin Mix^g^	1.00	1.00	1.00	1.00	—
Choline chloride	0.30	0.30	0.30	0.30	—
Starch	0.30	1.10	1.12	1.14	—
Taurine	0.10	0.15	0.18	0.21	—
Lysine	—	0.35	0.60	0.85	—
Methionine	—	0.30	0.50	0.70	—
Monocalcium phosphate	—	0.50	1.00	1.50	—
Carophyll pink 10%	0.10	0.10	0.10	0.10	—
*Proximate composition*					
Dry matter	93.8	93.7	94.5	94.2	91.5
Protein	52.4	54.1	52.1	52.0	54.8
Lipid	21.3	21.8	21.3	21.8	7.5
Ash	11.2	10.8	10.2	9.5	15.5

^a^Blumar fish meal, Chile.

^b^Aker Biomarine, Norway.

^c^Shandong Wonderful Biotech Co., Ltd. Dongying, China.

^d^SCI Co., Ltd. Chungcheongnam-do, Korea.

^e^Daekyung Oil & Transportation Co., Ltd. Busan, Korea.

^f^Mineral mixture composition (g/kg mix); ferrous fumarate, 12.5; manganese sulfate, 11.3, ferrous sulfate, 20; cupric sulfate, 1.25; cobaltous sulfate, 0.75; zinc sulfate, 13.75; calcium iodate, 0.75; magnesium sulfate, 80.2; aluminum hydroxide, 0.75.

^g^Vitamin mixture composition (unit/kg mix): ascorbic acid, 6400 mg; tocopherol acetate, 37,500 mg; thiamin nitrate, 5000 mg; riboflavin, 10,000 mg; pyridoxine hydrochloride, 5000 mg; nicotinic acid, 37,500 mg; Ca-D-pantothenate, 17,500 mg; inositol, 75,000 mg; biotin, 50 mg; folic acid, 2500 mg; menadione sodium bisulfite, 2500 mg; retinol acetate, 5,000,000 IU; cholecalciferol, 1,000,000 IU; cyanocobalamin, 25 mg; riboflavin, 10,000 mg.

**Table 2:** Amino acid composition of experimental diets (percentage of total AA).

Amino acids	FM60	FM45	FM30	FM15	COMF
Essential amino acids					
Arg	6.29	6.34	6.32	6.47	6.19
His	2.40	2.31	2.22	2.13	3.02
Ile	4.03	3.75	3.60	3.62	4.10
Met	2.94	3.28	3.51	3.46	2.82
Leu	8.50	8.02	7.73	7.43	8.11
Lys	7.42	7.55	7.27	7.34	7.58
Phe	4.36	4.16	4.13	4.11	4.29
Thr	4.50	4.32	4.02	3.84	4.49
Val	4.65	4.53	4.37	4.40	4.68
Nonessential amino acids					
Asp	10.17	9.82	9.42	9.35	9.91
Ser	4.66	4.50	4.49	4.35	4.40
Glu	16.22	15.72	15.66	15.49	15.18
Gly	6.28	7.19	7.67	8.17	6.96
Ala	6.37	6.39	6.23	6.06	6.64
Cys	0.94	0.92	1.00	0.96	0.80
Pro	5.10	5.64	6.39	6.67	5.33
Hypro	0.74	1.47	1.93	2.23	1.27
Tyr	3.03	2.79	2.78	2.62	2.94

**Table 3:** Fatty acid profiles and lipid nutritional quality indices of experimental diets (percentage of total fatty acids).

Fatty acids	FM60	FM45	FM30	FM15	COMF
6:0	0.02	0.04	0.05	0.06	0.00
8:0	0.00	0.00	0.00	0.02	0.00
10:0	0.00	0.02	0.02	0.02	0.05
12:0	0.07	0.07	0.08	0.08	0.17
13:0	0.01	0.01	0.01	0.01	0.05
14:0	3.87	3.44	3.27	3.14	4.12
14:1	0.04	0.06	0.07	0.08	0.11
15:0	0.20	0.18	0.17	0.16	0.72
16:0	17.23	17.09	16.88	16.83	22.08
16:1	4.23	3.97	3.81	3.69	4.97
17:0	0.18	0.19	0.20	0.20	0.91
18:0	4.15	4.61	4.78	4.96	7.20
18:1 n-9 trans	0.16	0.23	0.26	0.31	0.54
18:1 n-9 cis	25.48	27.30	28.15	29.25	20.55
18:2 n-6 trans	0.35	0.36	0.36	0.35	0.23
18:2 n-6 cis	25.91	25.90	25.85	25.97	8.68
20:0	0.29	0.28	0.27	0.27	0.41
18:3 n-6	0.12	0.11	0.11	0.11	0.14
20:1	2.22	1.99	1.79	1.62	1.35
18:3 n-3	3.40	3.39	3.38	3.38	1.46
21:0	0.04	0.05	0.06	0.07	0.08
20:2	0.35	0.36	0.36	0.37	0.32
22:0	0.24	0.25	0.25	0.25	0.29
20:3, n-6	0.14	0.15	0.16	0.16	0.18
22:1 n-9	0.36	0.33	0.31	0.31	0.23
20:3 n-3	0.08	0.08	0.08	0.08	0.11
23:0	0.03	0.03	0.03	0.04	0.11
20:4 n-6	0.25	0.28	0.29	0.30	1.63
22:2	0.37	0.00	0.32	0.31	0.00
24:0	0.13	0.13	0.12	0.12	0.40
20:5 n-3	5.61	5.05	4.71	4.19	6.46
24:1	0.56	0.46	0.39	0.30	0.99
22:6 n-3	3.90	3.60	3.39	2.99	15.44
∑SFA	26.37	26.25	26.04	26.05	36.32
∑MUFA	33.01	34.27	34.71	35.48	28.63
∑n3FA	13.00	12.12	11.56	10.63	23.47
∑n6FA	26.76	26.81	26.77	26.90	10.87
∑HUFA	9.98	9.15	8.63	7.71	23.83
∑n3/∑n6	0.49	0.45	0.43	0.40	2.16

**Table 4:** Growth performance, feed utilization, and survival of chum salmon (*Oncorhynchus keta*) fed the five experimental diets for 6 weeks.

Diets	FBW (g)^a^	WG (%)^b^	SGR (%)^c^	FI (g/fish)^d^	FE (%)^e^	PER^f^	SUR (%)^g^
FM60	15.0 ± 0.42^1,2^	150 ± 6.05	2.23 ± 0.06	15.0 ± 0.89^1^	60.6 ± 2.64	1.16 ± 0.05	72.7 ± 9.68
FM45	15.4 ± 0.89^1,2^	152 ± 20.4	2.14 ± 0.18	15.5 ± 0.18^1^	59.6 ± 5.80	1.16 ± 0.07	68.7 ± 8.97
FM30	16.4 ± 0.20^1^	176 ± 6.66	2.36 ± 0.06	14.5 ± 0.73^1^	72.3 ± 3.66	1.39 ± 0.07	80.0 ± 8.33
FM15	15.9 ± 1.15^1^	177 ± 23.2	2.35 ± 0.19	14.4 ± 0.21^1,2^	70.6 ± 7.72	1.36 ± 0.15	75.0 ± 12.3
COMF	13.5 ± 0.31^2^	145 ± 16.4	2.07 ± 0.15	12.6 ± 0.40^2^	63.3 ± 5.07	1.15 ± 0.09	72.0 ± 1.63

*Note*: Values are the mean of triplicate groups and are presented as mean ± SE. Values with different superscripts in the same row are significantly different (*p* < 0.05). The lack of superscript numbers indicates no significant differences among treatments.

^a^Final mean body weight.

^b^Weight gain percentage = 100 × (final mean body weight − initial mean body weight)/initial mean body weight.

^c^Specific growth rate = ([log_e_ final body weight − log_e_ initial body weight]/days) × 100.

^d^Feed intake = dry feed consumed (g)/number of fish.

^e^Feed efficiency = (fish wet weight gain/feed intake) × 100.

^f^Protein efficiency ratio = wet weight gain/total protein given.

^g^Survival rate = (survival fish number/total fish number) × 100.

**Table 5:** Nonspecific immune response of chum salmon (*Oncorhynchus keta*) fed the five experimental diets for 6 weeks.

Diets	Lysozyme^a^	SOD^b^
FM60	115 ± 7.04	71.5 ± 1.87
FM45	119 ± 2.61	73.1 ± 2.16
FM30	120 ± 7.19	70.9 ± 2.92
FM15	118 ± 5.71	72.6 ± 3.67
COMF	116 ± 4.17	79.1 ± 2.38

*Note*: Values are means of triplicate groups and presented as mean ± SE.

^a^Lysozyme activity (*µ*g mL^−1^).

^b^Superoxide dismutase (% inhibition).

**Table 6:** Plasma biochemical parameters of chum salmon (*Oncorhynchus keta*) fed the five experimental diets for 6 weeks.

Diets	GOT^a^	GPT^b^	ALP^c^	T-CHO^d^	TG^e^	TP^f^	GLU^g^
FM60	526 ± 16.2	22.0 ± 1.15	270 ± 16	204 ± 36	442 ± 29	5.30 ± 0.26	145 ± 6.01
FM45	478 ± 64.3	20.7 ± 1.86	217 ± 26	212 ± 32	287 ± 14	4.87 ± 0.20	155 ± 5.29
FM30	531 ± 43.1	21.0 ± 0.58	240 ± 58	236 ± 33	312 ± 59	5.07 ± 0.38	152 ± 26.9
FM15	521 ± 53.4	23.7 ± 0.67	290 ± 43	292 ± 77	421 ± 79	5.87 ± 0.47	177 ± 2.19
COMF	532 ± 11.3	22.7 ± 2.19	273 ± 46	321 ± 28	423 ± 77	5.87 ± 0.73	140 ± 5.21

*Note*: Values are the mean of triplicate groups and are presented as mean ± SE.

^a^Glutamic-oxaloacetic transaminase (U/L).

^b^Glutamic-Pyruvic Transaminase (U/L).

^c^Alkaline phosphatase (U/L).

^d^Total cholesterol (mg/dL).

^e^Triglycerides (ng/dL).

^f^Total protein (g/dL).

^g^Glucose (mg/dL).

**Table 7:** Biometric parameters of chum salmon (*Oncorhynchus keta*) fed the five experimental diets for 6 weeks.

Diets	Condition factor^a^	Hepatosomatic index^b^	Viscerosomatic index^c^
FM60	0.78 ± 0.00^2^	1.76 ± 0.13	9.78 ± 0.74
FM45	0.81 ± 0.02^1,2^	1.54 ± 0.13	9.19 ± 0.05
FM30	0.79 ± 0.02^1,2^	1.75 ± 0.28	9.70 ± 0.87
FM15	0.84 ± 0.02^1^	1.58 ± 0.04	10.5 ± 0.52
COMF	0.77 ± 0.02^2^	1.77 ± 0.13	9.46 ± 0.40

*Note*: Values are the mean of triplicate groups and are presented as mean ± SE. Values with different superscripts in the same raw are significantly different (*p* < 0.05). The lack of superscript numbers indicates no significant differences among treatments.

^a^Condition factor = weight (g)×100/length^3^ (cm).

^b^Hepatosomatic index = (liver weight (g)/fish weight (g)×100.

^c^Viscerosomatic index = (Viscera weight (g)/fish weight (g)×100.

**Table 8:** Muscle proximate composition (wet basis [%]) of chum salmon (*Oncorhynchus keta*) fed the five experimental diets for 6 weeks.

Diets	Dry matter	Protein	Lipid	Ash
FM60	25.5 ± 0.49	21.6 ± 0.65	2.06 ± 0.35	1.36 ± 0.05
FM45	24.2 ± 0.47	22.3 ± 1.13	1.73 ± 0.53	1.44 ± 0.05
FM30	24.3 ± 0.07	21.1 ± 0.14	1.45 ± 0.34	1.38 ± 0.04
FM15	25.8 ± 0.32	20.9 ± 0.71	2.12 ± 0.18	1.34 ± 0.06
COMF	26.0 ± 0.69	21.6 ± 0.34	1.75 ± 0.08	1.35 ± 0.04

*Note*: Values are the mean of triplicate groups and are presented as mean ± SE.

**Table 9:** Muscle fatty acid profiles and lipid nutritional quality indices of chum salmon (*Oncorhynchus keta*) fed the five experimental diets for 6 weeks (percentage of total fatty acids).

Fatty acids	FM60	FM45	FM30	FM15	COMF
14:0	2.36 ± 0.23	2.32 ± 0.13	2.13 ± 0.16	2.29 ± 0.09	2.51 ± 0.35
15:0	0.19 ± 0.02^2^	0.18 ± 0.00^2^	0.19 ± 0.00^2^	0.17 ± 0.00^2^	0.40 ± 0.03^1^
16:0	17.2 ± 0.39^1,2^	16.8 ± 0.62^2,3^	16.9 ± 0.25^2,3^	16.2 ± 0.50^3^	17.9 ± 0.31^1^
16:1	3.07 ± 0.19^2^	3.10 ± 0.07^2^	2.87 ± 0.16^2^	3.06 ± 0.10^2^	4.17 ± 0.49^1^
17:0	0.22 ± 0.01^2^	0.21 ± 0.01^2^	0.22 ± 0.00^2^	0.20 ± 0.01^2^	0.45 ± 0.03^1^
18:0	4.86 ± 0.21^2^	4.88 ± 0.13^2^	5.00 ± 0.14^2^	4.83 ± 0.13^2^	5.66 ± 0.20^1^
18:1 n-9 trans	0.87 ± 0.34	0.73 ± 0.04	0.79 ± 0.08	0.81 ± 0.03	1.00 ± 0.02
18:1 n-9 cis	22.3 ± 0.83^3^	23.7 ± 0.85^2,3^	23.4 ± 1.01^2,3^	26.3 ± 0.68^1^	24.6 ± 1.90^1,2^
18:2 n-6 trans	0.29 ± 0.12^1,2^	0.30 ± 0.02^1,2^	0.29 ± 0.12^1,2^	0.34 ± 0.03^1^	0.17 ± 0.00^2^
18:2 n-6 cis	19.5 ± 1.72^2^	20.6 ± 0.77^2^	19.7 ± 0.20^2^	22.8 ± 1.15^1^	8.80 ± 0.91^3^
20:0	0.14 ± 0.01	0.15 ± 0.00	0.09 ± 0.08	0.14 ± 0.00	0.09 ± 0.08
18:3 n-6	0.27 ± 0.03^3^	0.34 ± 0.01^2^	0.35 ± 0.03^2^	0.46 ± 0.02^1^	0.16 ± 0.01^4^
20:1	1.58 ± 0.10^1,2^	1.54 ± 0.06^2,3^	1.36 ± 0.05^3^	1.36 ± 0.04^3^	1.73 ± 0.18^1^
18:3 n-3	2.34 ± 0.09^2^	2.41 ± 0.12^1,2^	2.30 ± 0.06^2^	2.53 ± 0.10^1^	1.43 ± 0.04^3^
20:2	0.78 ± 0.05^1^	0.83 ± 0.04^1^	0.80 ± 0.00^1^	0.82 ± 0.06^1^	0.64 ± 0.04^2^
20:3, n-6	0.55 ± 0.07^3^	0.60 ± 0.02^2,3^	0.64 ± 0.02^1,2^	0.71 ± 0.03^1^	0.40 ± 0.02^4^
22:1 n-9	0.20 ± 0.03^2^	0.19 ± 0.02^2^	0.19 ± 0.02^2^	0.19 ± 0.00^2^	0.27 ± 0.04^1^
20:3 n-3	0.07 ± 0.06	0.12 ± 0.01	0.08 ± 0.07	0.11 ± 0.00	0.10 ± 0.08
20:4 n-6	0.84 ± 0.02^2,3^	0.87 ± 0.06^2,3^	1.00 ± 0.08^2^	0.78 ± 0.10^3^	1.48 ± 0.15^1^
22:2	0.56 ± 0.02	0.18 ± 0.32	0.33 ± 0.29	0.33 ± 0.28	0.62 ± 0.06
20:5 n-3	4.11 ± 0.20^2^	3.88 ± 0.28^2^	3.95 ± 0.29^2^	3.13 ± 0.29^3^	4.69 ± 0.44^1^
24:1	0.54 ± 0.05^2^	0.47 ± 0.02^3^	0.46 ± 0.03^3^	0.35 ± 0.01^4^	0.62 ± 0.04^1^
22:6 n-3	16.9 ± 2.70^2^	15.2 ± 1.47^2^	16.6 ± 1.78^2^	11.7 ± 1.63^3^	21.6 ± 2.60^1^
∑SFA	25.0 ± 0.89^2^	24.7 ± 0.88^2^	24.6 ± 0.48^2^	24.0 ± 0.68^2^	27.1 ± 0.54^1^
∑MUFA	28.5 ± 0.62^3^	29.7 ± 0.94^2,3^	29.1 ± 1.21^3^	32.1 ± 0.8^1,2^	32.4 ± 2.52^1^
∑n3FA	23.4 ± 2.70^2^	21.6 ± 1.56^2^	23.0 ± 1.85^2^	17.4 ± 1.81^3^	27.9 ± 2.80^1^
∑n6FA	21.5 ± 1.68^2^	22.7 ± 0.74^2^	21.9 ± 0.05^2^	25.1 ± 0.96^1^	11.1 ± 1.03^3^
∑HUFA	22.5 ± 2.83^2^	20.6 ± 1.72^2,3^	22.3 ± 1.98^2^	16.4 ± 2.02^3^	28.3 ± 3.00^1^
∑n3/∑n6	1.10 ± 0.20^2^	0.95 ± 0.09^2,3^	1.04 ± 0.08^2,3^	0.69 ± 0.09^3^	2.52 ± 0.32^1^

*Note*: Values are the mean of triplicate groups and are presented as mean ± SE. Values with different superscripts in the same raw are significantly different (*p* < 0.05). The lack of superscript numbers indicates no significant differences among treatments.

**Table 10:** Muscle amino acid composition of chum salmon (*Oncorhynchus keta*) fed the five experimental diets for 6 weeks (percentage per total AA).

Amino acids	FM60	FM45	FM30	FM15	COMF
Essential amino acids					
Arg	5.91 ± 0.03	5.81 ± 0.06	5.83 ± 0.02	5.83 ± 0.06	5.88 ± 0.03
His	2.99 ± 0.11	3.14 ± 0.14	3.14 ± 0.11	3.00 ± 0.16	3.06 ± 0.10
Ile	4.13 ± 0.12	4.06 ± 0.42	4.07 ± 0.44	4.18 ± 0.40	4.07 ± 0.13
Met	3.24 ± 0.17	3.69 ± 0.25	3.60 ± 0.26	3.53 ± 0.30	3.46 ± 0.16
Leu	8.55 ± 0.11	8.39 ± 0.17	8.41 ± 0.23	8.49 ± 0.22	8.46 ± 0.17
Lys	9.24 ± 0.13	8.93 ± 0.58	8.98 ± 0.55	9.00 ± 0.59	9.16 ± 0.16
Phe	4.69 ± 0.01	4.77 ± 0.04	4.77 ± 0.01	4.68 ± 0.08	4.72 ± 0.04
Thr	5.21 ± 0.17	5.32 ± 0.21	5.28 ± 0.24	5.22 ± 0.25	5.27 ± 0.15
Val	5.01 ± 0.02	4.82 ± 0.47	4.80 ± 0.44	4.87 ± 0.37	4.94 ± 0.11
Nonessential amino acids					
Asp	9.94 ± 0.69	9.67 ± 0.69	9.82 ± 0.73	10.06 ± 0.8	9.74 ± 0.52
Ser	4.63 ± 0.12	4.52 ± 0.07	4.55 ± 0.14	4.53 ± 0.19	4.68 ± 0.06
Glu	16.1 ± 0.13	16.0 ± 0.35	15.9 ± 0.48	15.9 ± 0.35	16.1 ± 0.05
Gly	4.44 ± 0.10	4.35 ± 0.16	4.41 ± 0.17	4.47 ± 0.25	4.41 ± 0.12
Ala	6.40 ± 0.05	6.50 ± 0.09	6.47 ± 0.12	6.45 ± 0.16	6.44 ± 0.03
Cys	1.08 ± 0.08	1.03 ± 0.06	1.05 ± 0.09	1.05 ± 0.09	1.06 ± 0.08
Pro	3.65 ± 0.56	3.80 ± 1.16	3.84 ± 0.92	3.66 ± 1.06	3.61 ± 0.69
Tyr	3.16 ± 0.11	3.45 ± 0.30	3.43 ± 0.36	3.35 ± 0.34	3.30 ± 0.22

*Note*: Values are the mean of triplicate groups and are presented as mean ± SE.

## Data Availability

The data that support the findings of this study are available on request from the corresponding author. The data are not publicly available due to privacy or ethical restrictions.
